# Heart Failure–Associated Tension Chylothorax: A Rare Presentation of a Common Disease

**DOI:** 10.7759/cureus.94787

**Published:** 2025-10-17

**Authors:** Dhiraj R Regmi, Sabita Regmi, Pratick Shrestha, Laxman Wagle

**Affiliations:** 1 Internal Medicine, Nepalese Army Institute of Health Sciences, Kathmandu, NPL; 2 Internal Medicine, Tribhuvan University Institute of Medicine, Kathmandu, NPL; 3 Internal Medicine, Vayodha Hospital, Kathmandu, NPL; 4 Internal Medicine, Karnali Academy of Health Sciences, Karnali, NPL

**Keywords:** chylothorax, heart failure, heart failure with preserved ejection fraction (hfpef), pleural effusion, tension chylothorax

## Abstract

Heart failure can present along with pleural effusion, which is usually bilateral and biochemically transudative. This pleural effusion can rarely be chylous, indicating chylothorax. Herein, we describe a case of an 86-year-old male patient with heart failure with reduced ejection fraction who presented with a two-week history of progressive dyspnea and bilateral leg swelling. Physical findings included absent breath sounds in the right mid- and lower lung zones, left basilar crackles, and 3+ pitting edema up to the knees. Chest X-ray and computed tomography of the chest revealed a large right-sided pleural effusion with associated atelectasis. Emergent thoracentesis was performed, yielding 2 L of milky fluid. A pleural fluid triglyceride level of 414 mg/dL confirmed the diagnosis of chylothorax. A CT scan of the chest, abdomen, and pelvis with intravenous contrast and pleural fluid cytology did not show any findings suggestive of malignancy or infection. Blood and pleural fluid cultures were negative. The acid-fast bacilli (AFB) smear, culture, and interferon gamma release assay were unremarkable. Tumor markers, including CA 19-9, CEA, and AFP, were negative. A serum lipid panel was unremarkable. An echocardiogram showed a left ventricular ejection fraction of 40% with diffuse hypokinesis and pulmonary artery systolic pressure of 43 mmHg; the brain natriuretic peptide level was 1290 pg/mL, consistent with decompensated heart failure. The patient was treated with intravenous furosemide, and his symptoms significantly improved during his hospital stay. He was subsequently discharged with instructions for fluid and salt restriction and a low-fat diet with medium-chain triglycerides (MCTs); dietitian counseling was also provided.

A pleural fluid triglyceride level of more than 110 mg/dL can establish the diagnosis of chylothorax. In heart failure, the mechanism of chylothorax is likely increased hydrostatic pressure in the lymphatics due to volume overload and high venous pressure. In addition, high venous pressure, increasing the abdominal lymph production, is also implicated. Treatment of underlying heart failure with diuresis along with a low-fat diet with MCT supplementation usually results in improvement.

## Introduction

Chylothorax refers to the accumulation of chyle in the pleural space, typically resulting from obstruction or disruption of the thoracic duct [1]. Trauma, particularly iatrogenic injury, is the major contributor for the chylothorax, with few incidences of congenital and non-traumatic causes reported. Among these, malignancy is the leading non-traumatic cause, with approximately 70% of cases attributed to lymphoma. Other causes include sarcoidosis, congenital duct anomalies, cirrhosis, and lymphangioleiomyomatosis [2]. The pleural fluid in chylothorax is classically exudative, though rare instances of transudative chylothorax have been documented in conditions such as cirrhosis, congestive heart failure, and nephrotic syndrome [3]. Heart failure itself commonly presents with bilateral pleural effusions that are typically transudative in nature. Herein, we report a rare case of tension chylothorax in the setting of heart failure.

## Case presentation

**Figure 1:**
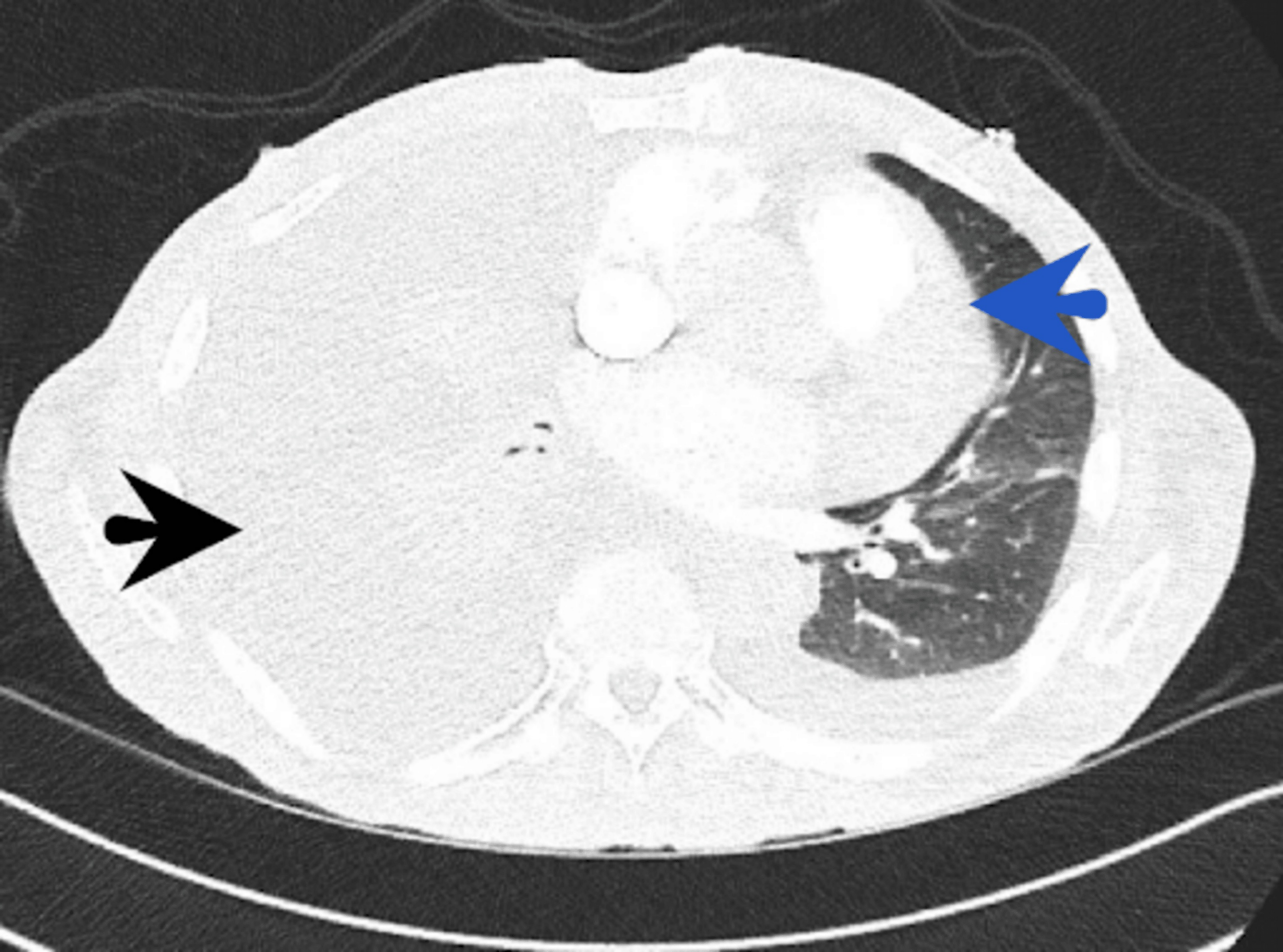
A CT scan of the chest confirmed a significant right-sided pleural effusion with a mediastinal shift to the left, along with features suggestive of tension hydrothorax (black arrow). A smaller left-sided pleural effusion was also noted (blue arrow).

An 86-year-old man with heart failure with reduced ejection fraction presented with a two-week history of dyspnea and bilateral leg swelling. He was tachypneic, with a respiratory rate of 28 breaths per minute. Notable physical findings included absent breath sounds in the right mid and lower lung zones, left basilar crackles, and 3+ pitting edema extending up to the knees. A chest X-ray revealed a large right-sided pleural effusion with associated atelectasis. A subsequent computed tomography (CT) scan of the chest confirmed a significant right-sided pleural effusion with mediastinal shift to the left, along with features suggestive of tension hydrothorax. A smaller left-sided pleural effusion was also noted (Figure 1).

**Figure 2:**
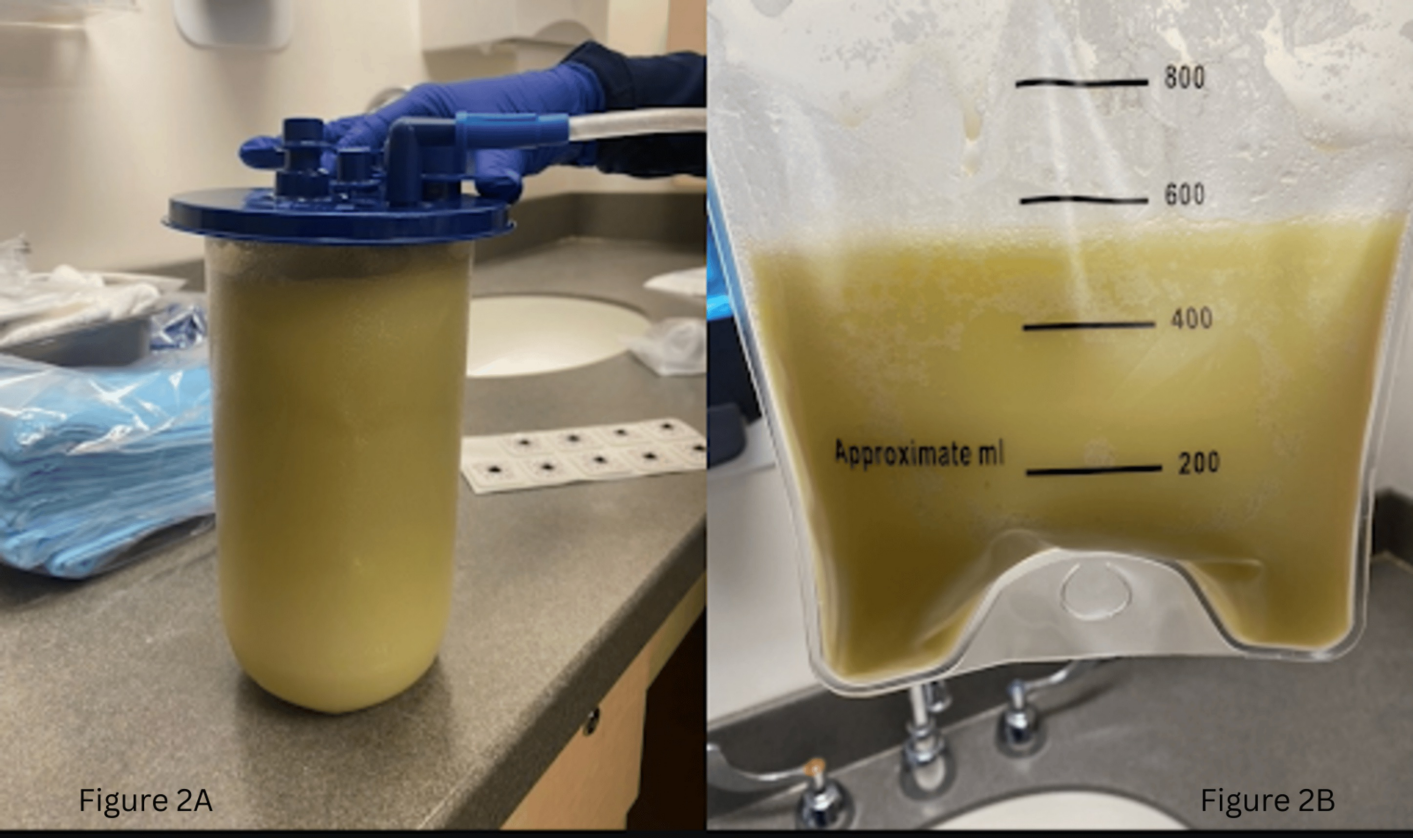
Emergent thoracentesis yielded 2 L of milky fluid raising concerns of chylothorax. Milky fluid in a suction cannister (2A) and the same milky fluid in a suction bag after its drainage (2B). The amount in the bag is noted to be around 500 mL whereas the cannister had 1.5 L of fluid.

Emergent right-sided thoracentesis was performed, yielding 2 L of milky fluid, raising suspicion for chylothorax (Figure 2). The pleural fluid did not meet Light’s criteria for exudative effusion and was deemed transudative. The pleural fluid triglyceride level was 414 mg/dL, confirming the diagnosis of chylothorax.

A CT scan of the chest, abdomen, and pelvis with intravenous contrast showed no evidence of malignancy or significant lymphadenopathy. Pleural fluid cytology showed a mixed population of neutrophils and lymphocytes, with no features suggestive of lymphoma. Flow cytometry of the pleural fluid could not be performed due to a low white blood cell count. Both blood and pleural fluid cultures were negative. Additionally, an acid-fast bacillus (AFB) smear, culture, and interferon gamma release assay were all unremarkable. Tumor markers, including CA 19-9, CEA, and AFP, were all within normal limits. A serum lipid panel showed a triglyceride level of 66 mg/dL and a total cholesterol level of 130 mg/dL. Laboratory results are summarized in Table 1.

**Table 1:** Laboratory work-up including complete blood count, comprehensive metabolic panel, pleural fluid analysis and serum lipid test results N/A: not available

Test	Value	Reference range
WBC	6.5 × 10³/µL	4.0–11.0 × 10³/µL
Hemoglobin	13.8 g/dL	13.5–17.5 g/dL
Hematocrit	41%	41%–53%
Platelets	210 × 10³/µL	150–450 × 10³/µL
Sodium	138 mmol/L	135–145 mmol/L
Potassium	4.2 mmol/L	3.5–5.0 mmol/L
Chloride	100 mmol/L	98–106 mmol/L
Bicarbonate (CO₂)	24 mmol/L	22–29 mmol/L
Blood urea nitrogen (BUN)	16 mg/dL	7–20 mg/dL
Creatinine	1.0 mg/dL	0.6–1.3 mg/dL
Glucose (random)	102 mg/dL	70–140 mg/dL
Aspartate aminotransferase	50 U/L	10–40 U/L
Alanine aminotransferase	45 U/L	7–56 U/L
Alkaline phosphatase	90 U/L	44–147 U/L
Total bilirubin	0.8 mg/dL	0.2–1.2 mg/dL
Direct bilirubin	0.2 mg/dL	0–0.3 mg/dL
Total protein	7.2 g/dL	6.0–8.3 g/dL
Albumin	4.3 g/dL	3.4–5.4 g/dL
Globulin	2.9 g/dL	2.0–3.5 g/dL
Pleural fluid appearance	Milky	Clear/straw-colored
Light’s criteria	Transudative	N/A
Pleural fluid triglycerides	414 mg/dL	<50 mg/dL
Pleural fluid cell differential	Mixed neutrophils and lymphocytes	N/A
Pleural fluid cytology	Negative for malignancy	N/A
Acid-fast bacillus (AFB) smear/culture	Negative	Negative
Pleural fluid culture	Negative	Negative

An echocardiogram revealed a left ventricular ejection fraction of 40%, with diffuse hypokinesis and a pulmonary artery systolic pressure of 43 mmHg. Given the elevated brain natriuretic peptide level of 1290 pg/mL, bilateral leg swelling, and evidence of heart failure with reduced ejection fraction, the chylothorax was most likely secondary to heart failure.

The patient was treated with intravenous furosemide to alleviate fluid retention, and his symptoms significantly improved during his hospital stay. He was discharged with instructions for fluid and salt restriction, and a low-fat diet combined with medium-chain triglycerides (MCTs). Dietary education was provided by a dietitian.

## Discussion

Chyle is a lymphatic fluid enriched with fat and its digestive products absorbed by the intestinal epithelium [4]. Chylothorax is the presence of chyle in the pleural space; it is extremely rare, accounting for only 3% of the pleural effusion [5]. Direct detection of chylomicrons via lipoprotein electrophoresis is considered the gold standard for diagnosing chylothorax; however, it is infrequently performed in clinical practice due to its cost, limited availability, and technical complexity. Instead, a pleural fluid triglyceride level greater than 110 mg/dL and a cholesterol level less than 200 mg/dL are considered highly suggestive of chylothorax [6,7]. Triglyceride levels ranging from 50 to 110 mg/dL are indeterminate, and a triglyceride level less than 50 mg/dL rules out chylothorax. In a retrospective study by Maldonado et al., the classical milky appearance of pleural fluid was observed in only 44% of chylothorax cases, while serous and serosanguinous appearances were equally common [1]. Therefore, pleural fluid analysis must be considered whenever there is suspicion of chylothorax, irrespective of the physical appearance of the pleural fluid.

In most cases, chylothorax is exudative, and transudative chylothorax is considered a rare entity. Notably, approximately 14% to 32% of reported chylothorax cases have been transudative in nature. In such instances, the underlying causes are typically systemic conditions that alter hydrostatic or oncotic pressures, rather than direct lymphatic injury or inflammation. These include conditions commonly associated with transudative pleural effusions, such as congestive heart failure, hepatic cirrhosis, constrictive pericarditis, nephrotic syndrome, superior vena cava obstruction, and amyloidosis [1,3]. In our case, the chylothorax was transudative and was, in our clinical context, secondary to congestive heart failure.

In heart failure, the development of chylothorax is likely due to increased hydrostatic pressure within the lymphatic system, particularly the thoracic duct, caused by volume overload and elevated central venous pressure [6]. Additionally, increased pressure in the left subclavian vein can impede lymphatic drainage, ultimately leading to chyle leakage [8]. The transudative nature of heart-failure-associated chylothorax is likely due to heart failure producing fluid with transudative characteristics, which then mixes with the chyle [6].

Chylothorax typically presents with symptoms similar to other pleural effusions, such as dyspnea, cough, and chest discomfort. However, it usually does not cause pleuritic chest pain or fever, as chyle is non-irritant [4,9]. In contrast, post-traumatic rapid accumulation of chyle can lead to respiratory and hemodynamic collapse, resulting in tension chylothorax [6,10]. There are no documented cases of tension chylothorax occurring as a direct consequence of heart failure. However, tension chylothorax has occasionally been reported following pneumonectomy, where the gradual accumulation of large volumes of chyle leads to the mediastinal shift and eventual hemodynamic compromise [6]. A similar mechanism may have been at play in our patient, who developed symptoms progressively over two weeks, ultimately resulting in hemodynamic collapse. In the setting of an atypical pleural effusion, testing for natriuretic peptides such as N-terminal pro-B-type natriuretic peptide (NT-proBNP) can aid in diagnosing or ruling out heart failure [11].

In patients with non-traumatic chylothorax presenting with large, symptomatic pleural effusions, therapeutic interventions such as thoracocentesis, indwelling pleural catheter (IPC) insertion, or intercostal chest drain (ICD) placement may be considered [6]. Emergent tube thoracostomy is mandatory to relieve tension and improve venous return and alleviate respiratory and hemodynamic compromise in the case of tension chylothorax [10].

The primary focus in managing chylothorax secondary to heart failure should be optimization of the underlying cardiac condition. This includes the use of diuretics to relieve volume overload, along with evidence-based therapies aimed at improving the overall cardiac function using angiotensin-converting enzyme (ACE) inhibitors or angiotensin receptor blockers (ARBs), sodium-glucose co-transporter 2 inhibitors (SGLT2 inhibitors), mineralocorticoid receptor antagonist (MRA), and beta-blockers. In rare cases where pleural effusions are refractory to medical therapy, additional interventions may be considered. These include pleurodesis to prevent recurrent fluid accumulation, or, as a last resort, the placement of an IPC for symptom control [11].

Simultaneously, addressing the chyle leak itself is essential. This is typically achieved by reducing lymphatic flow through dietary modifications, including a high-protein, low-fat diet supplemented with MCTs. Unlike long-chain triglycerides, MCTs are absorbed directly into the portal venous system, bypassing the intestinal lymphatics, thereby significantly reducing chyle production. In many cases, such dietary interventions lead to a substantial clinical improvement and may even promote spontaneous resolution of the chylothorax [6,11,12].

## Conclusions

Even though heart failure classically presents with bilateral transudative pleural effusion, it can rarely present with transudative chylothorax. Delay in diagnosis or prolonged decompensation increases the risk of progression to tension chylothorax. Hence, early consideration in the differential diagnosis, prompt recognition through targeted pleural fluid analysis, and emergent drainage in tension scenarios are critical. A combined strategy of optimizing cardiac function and reducing chyle flow with dietary modification can achieve resolution and often decreases the need for invasive procedures.
